# Cerebrospinal fluid drainage and chronic hydrocephalus in aneurysmal subarachnoid hemorrhage patients with intraventricular hemorrhage

**DOI:** 10.3389/fneur.2023.1302622

**Published:** 2023-12-15

**Authors:** Botao Wu, Yang Zhou, Hongjun Fan, Zhimin Liu, Wanyun Wu, Zebo Chen, Yong Yan, Wen Yuan, Wei Luo

**Affiliations:** ^1^Department of Neurosurgery, The Affiliated Zhuzhou Hospital of Xiangya Medical College, Central South University, Zhuzhou, Hunan, China; ^2^Loudi Vocational and Technical College, Loudi, Hunan, China

**Keywords:** aneurysmal subarachnoid hemorrhage (aSAH), intraventricular hemorrhage (IVH), chronic hydrocephalus, cerebrospinal fluid drainage, risk factors

## Abstract

**Background:**

Patients with intraventricular hemorrhage (IVH) are at a higher risk of developing hydrocephalus and often require external ventricular drainage or long-term ventriculoperitoneal shunt surgery.

**Objective:**

To investigate whether cerebrospinal fluid drainage in patients with IVH due to aneurysmal subarachnoid hemorrhage (aSAH) reduces the incidence of chronic hydrocephalus.

**Method:**

A retrospective analysis was conducted on patients with aSAH treated at our hospital between January 2020 and December 2022. The first analysis compared patients with and without IVH, while the second analysis compared IVH patients with and without chronic hydrocephalus. The third analysis compared IVH patients who underwent in different drainage methods which is lumbar drainage (LD) or external ventricular drainage (EVD). The primary outcome measure was the incidence of chronic hydrocephalus.

**Result:**

Of the 296 patients hospitalized with aSAH, 108 (36.5%) had IVH, which was associated with a significantly higher incidence of chronic hydrocephalus compared to patients without IVH (49.1% vs. 16.5%, *p* < 0.001). Multivariate logistic regression analysis showed that IVH was independently associated with the formation of chronic hydrocephalus (OR: 3.530, 95% CI: 1.958–6.362, *p* < 0.001). Among the 108 IVH patients, 53 (49.1%) developed chronic hydrocephalus. Multivariate logistic regression analysis revealed that the Hunt Hess grade at admission (OR: 3.362, 95% CI: 1.146–9.863, *p* = 0.027) and postoperative cerebrospinal fluid drainage (OR: 0.110, 95% CI: 0.036–0.336, *p* < 0.001) were independent risk factors for the development of chronic hydrocephalus in IVH patients. Among all IVH patients who underwent cerebrospinal fluid drainage, 45 (75%) received continuous lumbar puncture drainage, and 15 (25%) received external ventricular drainage. Univariate analysis did not show a statistically significant difference between the two groups in terms of postoperative chronic hydrocephalus (*p* = 0.283). However, multivariate logistic regression analysis suggested that the drainage methods of LD and EVD might be associated with the development of chronic hydrocephalus.

**Conclusion:**

The presence of IVH increases the risk of chronic hydrocephalus in patients with aSAH, and postoperative cerebrospinal fluid drainage appears to reduce this risk. The specific effects of lumbar puncture drainage and ventricular drainage on the incidence of chronic hydrocephalus require further investigation.

## Introduction

Aneurysmal subarachnoid hemorrhage (SAH) is a devastating disease that causes extensive bleeding into the subarachnoid space in a short period, often complicated by the diffusion of blood into the ventricular system. Approximately 30%–70% of cases of aneurysmal subarachnoid hemorrhage (aSAH) are accompanied by intraventricular hemorrhage (IVH). Previous studies have shown a close relationship between IVH and the formation of hydrocephalus in patients with aSAH (1–3).

Hydrocephalus is a common complication that can occur after an aneurysmal subarachnoid hemorrhage (aSAH), which refers to the accumulation of excessive cerebrospinal fluid in the ventricular system of the brain. The incidence rate of hydrocephalus in patients with aSAH varies between 6% and 67% (3–7). The development of hydrocephalus after aSAH is categorized into three stages based on the time elapsed since the onset of the hemorrhage: acute (0–3 days), subacute (4–13 days), and chronic (1–14 days before aSAH) (6, 8). While some patients may have self-limiting acute hydrocephalus, others may experience significant ventricular dilation and increased pressure within the brain, necessitating cerebrospinal fluid drainage to alleviate symptoms (9). The exact mechanism by which acute hydrocephalus progresses to chronic communicating hydrocephalus following subarachnoid hemorrhage is not yet fully understood. It is also important to note that not all patients with chronic hydrocephalus necessarily experience acute hydrocephalus. Several studies have implicated multiple mechanisms in the development of hydrocephalus after aSAH, including changes in cerebrospinal fluid dynamics, obstruction of arachnoid granules by blood products, and adhesions within the ventricular system (10). Numerous factors have been found to be associated with the occurrence of hydrocephalus after subarachnoid hemorrhage, including advanced age, hypertension, intraventricular hemorrhage, diffuse subarachnoid hemorrhage, aneurysms located in the posterior circulation, focal ischemic damage, ventricular enlargement upon admission, poor Hunt, Hess, and Fisher grading, symptomatic vasospasm, aneurysm rebleeding, and female gender (11). Van Gijn et al. reported that the extent of intraventricular hemorrhage is correlated with the development of hydrocephalus, whereas the location of cerebral hemorrhage and aneurysm rupture is not (12). Furthermore, in the absence of intraventricular hemorrhage, a higher volume of pool blood, particularly surrounding the brain, poses a risk factor for hydrocephalus. In summary, hydrocephalus can occur as a complication of aneurysmal subarachnoid hemorrhage. The development of hydrocephalus after aSAH involves multiple mechanisms, and numerous factors have been associated with its occurrence. Further research is required to fully understand the pathogenesis and risk factors involved, as well as to develop effective preventive and management strategies for hydrocephalus in patients with aSAH.

The removal of bloody cerebrospinal fluid from the subarachnoid space and cistern is crucial in the treatment of hydrocephalus associated with aneurysmal subarachnoid hemorrhage (aSAH). Early detection and placement of cerebrospinal fluid drainage devices can significantly improve functional outcomes and prevent further deterioration of function (13). Currently, the most commonly used methods for continuous drainage of bloody cerebrospinal fluid are external ventricular drainage (EVD) and lumbar spinal drainage (LD). The choice between EVD and LD depends on the clinical condition of the patient, with EVD typically preferred for patients with acute hydrocephalus or large amounts of intraventricular hemorrhage (IVH) (14). However, there is still no definitive standard for determining when to use LD or EVD after aSAH, and the optimal treatment method remains a subject of controversy and further investigation (15, 16). In particular, there is limited research on the differences between these two drainage methods in reducing the formation of hydrocephalus in patients with both aSAH and IVH. Therefore, the aim of this study is to identify the risk factors for hydrocephalus formation in aSAH patients with IVH and to compare the effects of EVD and LD in reducing the development of chronic hydrocephalus.

## Methods

### Patient identification and selection

We reviewed all patients with endovascular treatment between January 2020 to January 2022 in our institution. Inclusion criteria: aged 18–80 years; aSAH diagnosed by computed tomography (CT) or lumbar puncture in the medical center; aneurysm confirmed as the cause of SAH on digital subtraction angiography (DSA), three-dimensional CT angiography, or magnetic resonance angiography, which was the cause of the subarachnoid hemorrhage; endovascular therapy was performed. Exclusion criteria: intracranial aneurysm rupture caused by trauma and unexplained subarachnoid hemorrhage; microsurgical clipping surgery or conservative treatment; patients lost to follow-up.

### Clinical parameters

The baseline data of patients were recorded, including gender, age, smoking history, drinking history, hypertension history, diabetes history, coronary heart disease history, aneurysm rupture history, Hunt Hess classification, and GCS score at admission; Imaging features of aneurysms, such as aneurysm size (maximum diameter) and location; Whether there is postoperative cerebrospinal fluid drainage and the drainage method (continuous lumbar drainage or external ventricular drainage); Postoperative complications such as pulmonary infection, intracranial infection, hydrocephalus, and delayed cerebral ischemia (DCI). According to the imaging data at the time of discharge and the head CT scan followed up for 3 months after discharge, patients with hydrocephalus and those who require temporary or permanent intraventricular drainage catheters are considered to have chronic hydrocephalus. The interviewer turned a blind eye to this situation. DCI is defined as “cerebral infarction confirmed by CT or MRI or confirmed by autopsy after excluding surgical related infarction.” IVH is defined as Fisher Rating IV. Whether to perform cerebrospinal fluid drainage and what drainage method to use are all decided by the attending neurosurgeon. The interviewer was blinded to the condition.

This study has been approved by the Ethics Committee of The Affiliated Zhuzhou Hospital of Xiangya Medical College, Central South University.

### Outcome assessment

The primary outcome is chronic hydrocephalus. Hydrocephalus is defined as excessive cerebrospinal fluid in the ventricular system. The diagnosis of hydrocephalus is based on clinical manifestations and neuroimaging examinations. Hydrocephalus may manifest as headache, nausea, vomiting, coma, and/or gradual slowing of knowledge and motor activity, gait ataxia, cognitive impairment, and urinary incontinence (17). The diagnostic neuroimaging examination of hydrocephalus is calculated based on CT scans, the width of the third ventricle, and the value index of the internal media (CMI = B/A, where A is the width of the outer layer of the skull and B is the width of the lateral ventricles). CMI value higher than 0.25 and a third ventricular width greater than 7 millimeters are considered pathological (18). The diagnosis of hydrocephalus was confirmed by radiology and diagnosed by two experienced surgeons.

### Data analysis

**Figure 1:**
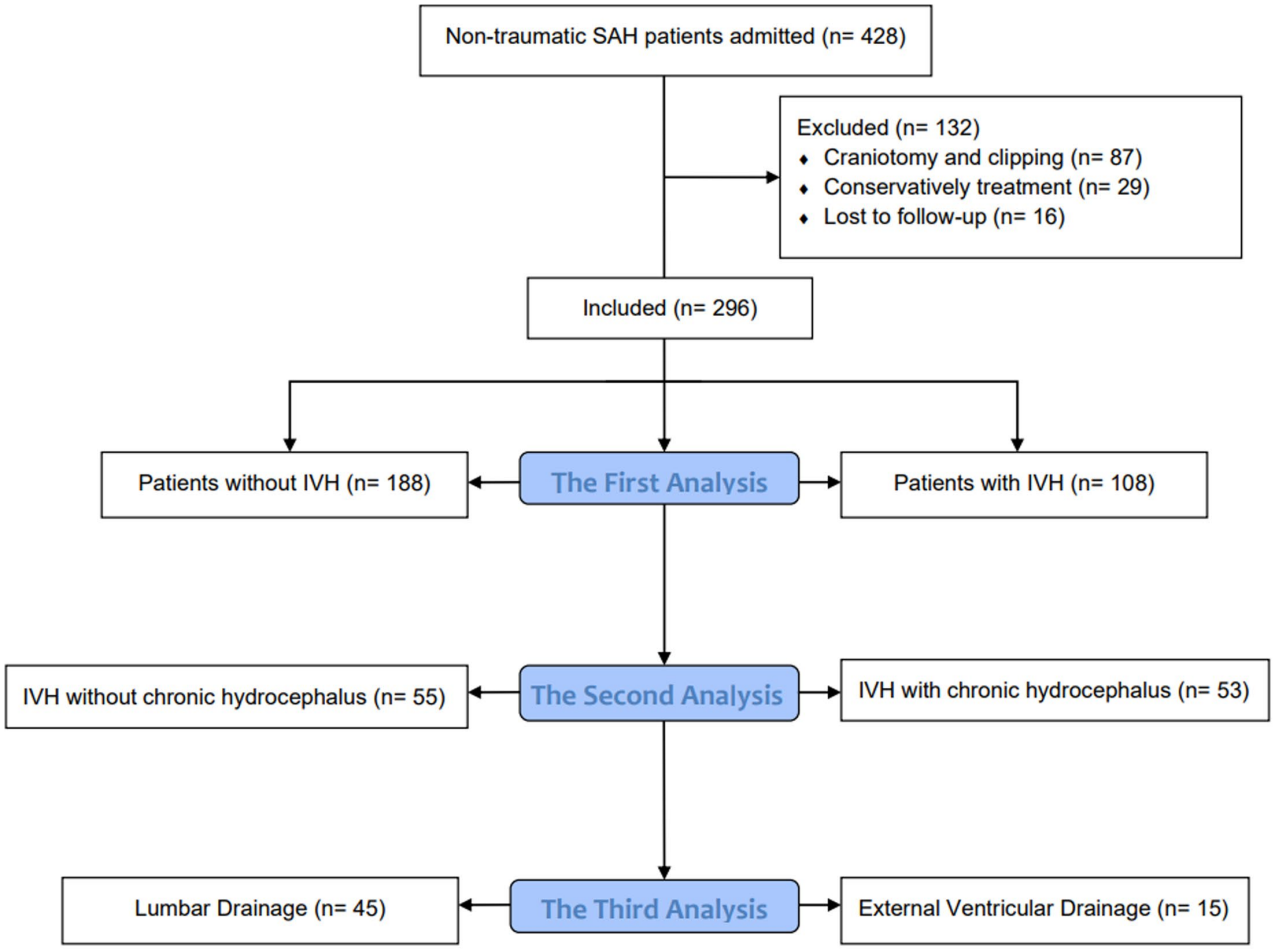
Flow diagram.

Statistical analysis using the SPSS 26.0 software (IBM, Armonk, NY). The measurement data conforming to the normal distribution is expressed as $\bar{x}$ ± s, the measurement data that is not normally distributed is expressed as the median and quartile [M (P25, P75)], and the comparison between groups is performed by *t*-test or rank sum test. Enumeration data were expressed as the number of cases and percentages [*n* (%)], and comparisons between groups were performed using the χ^2^ test or Fisher’s exact test. Divide patients into three queues for analysis. The first analysis compared patients with and without IVH, with the formation of chronic hydrocephalus as the dependent variable and the parameter *p* < 0.1 in the baseline data as the independent variable. Multivariate logistic regression analysis was used to adjust for inter-group differences in patients with or without IVH. The second analysis compared IVH patients with or without chronic hydrocephalus, with the presence or absence of chronic hydrocephalus as the dependent variable and the *p* < 0.1 parameter in the baseline data as the independent variable. Multivariate logistic regression analysis was used to analyze the influencing factors of chronic hydrocephalus formation in IVH patients. Finally, the third analysis compared the differences between groups of IVH patients with different cerebrospinal fluid drainage methods, and used multivariate logistic regression to analyze the influencing factors of chronic hydrocephalus formation in IVH patients with different drainage methods. *p* < 0.05 is defined as statistically significant (Figure 1).

## Results

### Baseline characteristics of the study cohort with or without intraventricular hemorrhage

A total of 296 hospitalized patients with aSAH were included in the study. As shown in Table 1, the incidence of IVH is 36.5% (*n* = 108). All IVH patients (*n* = 108) have a Fisher score of IV. IVH is associated with a higher incidence of hydrocephalus (49.1% vs. 16.5%, *p* < 0.001). There were statistically significant differences between the two groups in smoking history (*p* = 0.041), GCS score at admission (*p* < 0.001), Hunt Hess grade at admission (*p* = 0.001), postoperative cerebrospinal fluid drainage (*p* = 0.009), pulmonary infection (*p* < 0.001), and hydrocephalus (*p* < 0.001).

**Table 1:** Comparison of demographic, clinical, aneurysm, and treatment characteristics between patients with and without intraventricular hemorrhage.

	IVH		
Characteristic	YES (*n* = 108)	NO (*n* = 188)	*P*-value
Age, years, mean (SD)	60.9 (10.4)	59.7 (9.2)	0.307
Female, *n* (%)	72 (66.7)	129 (68.6)	0.729
Smoking history, *n* (%)	7 (6.5)	27 (14.4)	**0.041**
History of drinking alcohol, *n* (%)	10 (9.3)	18 (9.6)	0.929
Hypertension, *n* (%)	69 (63.9)	108 (57.4)	0.277
Heart disease, *n* (%)	8 (7.4)	21 (11.2)	0.294
Diabetes, *n* (%)	15 (13.9)	17 (9.0)	0.196
History of aSAH, *n* (%)	6 (5.6)	10 (5.3)	0.931
GCS score, M (P25, P75)	15 (10,15)	15 (15,15)	**<0.001**
Hunt Hess grade, M (P25, P75)	2 (1,3)	2 (1,2)	**0.001**
Aneurysm location			0.961
Anterior circulation, *n* (%)	91 (84.3)	158 (84.0)	
Posterior circulation, *n* (%)	17 (15.7)	30 (16.0)	
Aneurysm size (mm), mean (SD)	5.2 (1.7)	5.1 (2.0)	0.724
Aneurysm treatment time			0.550
<24 (h), *n* (%)	75 (69.4)	119 (63.3)	
24–72 (h), *n* (%)	26 (24.1)	53 (28.2)	
>72 (h), *n* (%)	7 (6.5)	16 (8.5)	
External cerebrospinal fluid (CSF)			**0.009**
No interventions, *n* (%)	48 (44.4)	113 (60.1)	
LD or EVD, *n* (%)	60 (55.6)	75 (39.9)	
Pulmonary infection, *n* (%)	51 (47.2)	36 (19.1)	**<0.001**
Intracranial infection, *n* (%)	14 (13.0)	12 (6.4)	**0.054**
Hydrocephalus, *n* (%)	53 (49.1)	31 (16.5)	**<0.001**
DCI, *n* (%)	6 (5.6)	3 (1.6)	**0.078**
Length of stay, mean (SD)	14.4 (7.3)	16.3 (11.5)	0.129

The value in bold indicates *p* < 0.1. aSAH, aneurysmal subarachnoid hemorrhage; IVH, intraventricular hemorrhage; SD, standard deviation; EVD, external ventricular drainage; LD, lumbar drainage; DCI, delayed cerebral ischemia; GCS, Glasgow Coma Scale. Divide patients into two groups based on the presence or absence of IVH. Groups were compared using the χ^2^ test, Fisher exact test, or Student *t*-test.

### Intraventricular hemorrhage is an independent risk factor for the formation of chronic hydrocephalus

Multivariate logistic regression analysis was used to adjust for the impact of differences between two groups of patients. The covariates in the multivariate model include smoking history, GCS score at admission, Hunt Hess grade at admission, pulmonary infection, intracranial infection, cerebrospinal fluid drainage, secondary ischemic stroke, and IVH. In multivariate logistic regression analysis, IVH was an independent risk factor for the development of chronic hydrocephalus (*p* < 0.001; Table 2).

**Table 2:** Multivariate logistic regression model adjustment for inter-group differences in patients with or without intraventricular hemorrhage.

Variable	OR	95%CI	*P*-value
Smoking history	0.714	0.253–2.013	0.525
GCS score	1.015	0.819–1.257	0.893
Hunt Hess grade	1.715	0.917–3.210	0.092
Pulmonary infection	1.541	0.812–2.926	0.186
External cerebrospinal fluid	0.884	0.468–1.671	0.705
Intracranial infection	2.190	0.798–6.006	0.128
DCI	1.326	0.275–6.390	0.725
IVH	3.530	1.958–6.362	**<0.001**

The values in bold are statistically significant (*P* < 0.05). IVH, intraventricular hemorrhage; DCI, secondary ischemic cerebral infarction; GCS, Glasgow Coma Scale; OR, Odds Ratio; CI, confidence interval.

### Intraventricular hemorrhage and chronic hydrocephalus

The study cohort included 108 patients with intraventricular hemorrhage, as shown in Table 3: 53 IVH patients had chronic hydrocephalus, and factors related to the formation of chronic hydrocephalus included GCS score at admission (*p* = 0.002), Hunt Hess grade at admission (*p* < 0.001), and postoperative cerebrospinal fluid drainage (*p* = 0.013).

**Table 3:** Comparison of demographic, clinical, aneurysm, and treatment characteristics between patients with and without hydrocephalus in intraventricular hemorrhage.

	Hydrocephalus		
Characteristic	YES (*n* = 53)	NO (*n* = 55)	*P*-value
Age, years, mean (SD)	60.4 (9.8)	61.4 (11.0)	0.636
Female, *n* (%)	31 (58.5)	41 (74.5)	**0.077**
Smoking history, *n* (%)	3 (5.7)	4 (7.3)	0.734
History of drinking alcohol, *n* (%)	5 (9.4)	5 (9.1)	0.951
Hypertension, *n* (%)	34 (64.2)	35 (63.6)	0.956
Heart disease, *n* (%)	3 (5.7)	5 (9.1)	0.716
Diabetes, *n* (%)	8 (15.1)	7 (12.7)	0.722
History of aSAH, *n* (%)	3 (5.7)	3 (5.5)	0.963
GCS score, mean (SD)	11.1 (3.5)	13.3 (3.6)	**0.002**
Hunt Hess grade, mean (SD)	2.7 (1.2)	1.8 (1.3)	**<0.001**
Aneurysm location			0.728
Anterior circulation, *n* (%)	44 (83.0)	47 (85.5)	
Posterior circulation, *n* (%)	9 (17.0)	8 (14.5)	
Aneurysm size (mm), mean (SD)	5.0 (1.8)	5.3 (1.6)	0.876
Aneurysm treatment time			0.276
24 (h), *n* (%)	33 (62.3)	42 (76.4)	
24–72 (h), *n* (%)	16 (30.2)	10 (18.2)	
>72 (h), *n* (%)	4 (7.5)	3 (5.5)	
External cerebrospinal fluid (CSF)			**0.013**
No interventions, *n* (%)	30 (56.6)	18 (32.7)	
LD or EVD, *n* (%)	23 (43.4)	37 (67.3)	
Pulmonary infection, *n* (%)	27 (50.9)	24 (43.6)	0.447
Intracranial infection, *n* (%)	7 (13.2)	7 (12.7)	0.941
DCI, *n* (%)	4 (7.5)	2 (3.6)	0.433
Length of stay, mean (SD)	17.6 (15.0)	15.0 (6.7)	0.236

The value in bold indicates *P* < 0.1. aSAH, aneurysmal subarachnoid hemorrhage; IVH, intraventricular hemorrhage; SD, standard deviation; EVD, external ventricular drainage; LD, lumbar drainage; DCI, delayed cerebral ischemia; GCS, Glasgow Coma Scale. Divide patients into two groups based on the presence or absence of chronic hydrocephalus. Groups were compared using the c2 test, Fisher exact test, or Student *t*-test.

### Factors influencing the formation of hydrocephalus in patients with intraventricular hemorrhage

Using logistic regression equations to identify the influencing factors of chronic hydrocephalus formation in patients with intraventricular hemorrhage, factors with a *p* value of less than 0.1 were included in the multivariate logistic regression equation, including gender, GCS score at admission, Hunt Hess grade at admission, and postoperative cerebrospinal fluid drainage. Hunt Hess grading at admission and postoperative cerebrospinal fluid drainage are independent risk factors for the formation of chronic hydrocephalus.

### IVH patients with lumbar drainage or external ventricular drainage

IVH patients who underwent postoperative cerebrospinal fluid drainage were compared according to different drainage methods. Patients who underwent lumbar drainage and ventricular drainage did not show statistical significance in the formation of chronic hydrocephalus (*p* = 0.283).

### Different drainage methods and the formation of chronic hydrocephalus

Although there was no statistically significant difference in the effectiveness of different cerebrospinal fluid drainage methods in reducing the formation of chronic hydrocephalus in patients with intraventricular hemorrhage during univariate analysis, and there was no significant difference in postoperative complications such as intracranial infection and DCI. However, when we included possible influencing factors and cerebrospinal fluid drainage methods in a multivariate logistic regression model, we found significant differences in GCS scores (*p* = 0.022) and the formation of chronic hydrocephalus (*p* = 0.003) between the two groups of patients at admission.

## Discussion

Chronic hydrocephalus after aSAH makes patients prone to worse neurological outcomes and cognitive impairment (3). The formation of hydrocephalus can affect prognosis, however, in current clinical practice, the accumulation of blood in the ventricular system is mainly used to assess the risk of cerebral vasospasm and DCI, often neglecting the risk of hydrocephalus formation. However, multiple studies have shown that the amount of blood in the cerebral cistern, ventricle, or brain parenchyma after subarachnoid hemorrhage, as well as acute hydrocephalus, is associated with the formation of chronic hydrocephalus in patients with aSAH (19, 20). Although the occurrence of hydrocephalus after aSAH has been well studied and some preventive measures have been taken, its potential risk factors are still unclear (21–23). Therefore, it is necessary to study the relationship between patients with intraventricular hemorrhage and the formation of chronic hydrocephalus, as well as how to reduce the incidence of chronic hydrocephalus.

Previous studies have shown that IVH is associated with hydrocephalus in patients with aSAH (24–26). The first analysis of this study found that the presence or absence of IVH at admission had a significant impact on the formation of hydrocephalus, with 108 out of 296 patients experiencing intraventricular hemorrhage (Table 1). The incidence of hydrocephalus in patients with intraventricular hemorrhage was almost three times higher than in patients without intraventricular hemorrhage (49.1% vs. 16.5%, *p* < 0.001). In contrast, patients with IVH have a more severe condition at admission (GCS score, *p* < 0.001; Hunt Hess grade, *p* = 0.001), which may be related to some patients experiencing acute hydrocephalus at admission, as IVH makes the cerebrospinal fluid circulation more easily blocked and the proportion of patients experiencing acute hydrocephalus is higher. According to our data, there is a difference in the proportion of postoperative cerebrospinal fluid drainage between the two groups of patients (55.6% vs. 39.9%, *p* = 0.009). This is also because the severity of the admitted patients’ condition is not consistent. We asked the neurosurgery surgeon, whether the patient should use cerebrospinal fluid drainage mainly depends on whether the patient has acute hydrocephalus, the degree of hydrocephalus, and the level of intracranial pressure. In the multivariate logistic regression equation, we adjusted for the impact of inter-group differences on the two groups of patients. Based on the results of statistical analysis, we believe that IVH is an independent risk factor for the formation of hydrocephalus in aSAH patients (OR = 3.530 [1.958–6.362], *p* < 0.001). The research results of Xu Hao et al. (27) also confirm this point.

The second analysis compared the risk factors for chronic hydrocephalus in all IVH patients. A total of 53 patients developed hydrocephalus. When comparing patients with and without hydrocephalus, we found that postoperative cerebrospinal fluid drainage had a significant effect on reducing the occurrence of hydrocephalus (*p* = 0.013). As shown in Table 3, among patients with hydrocephalus, 30 patients did not undergo postoperative cerebrospinal fluid drainage, while 23 patients underwent postoperative cerebrospinal fluid drainage (56.6% vs. 43.4%). Among patients without hydrocephalus, only 18 patients did not undergo postoperative cerebrospinal fluid drainage, and 37 patients underwent postoperative cerebrospinal fluid drainage (32.7% vs67.3%), There is a significant difference between the two (*p* = 0.013). Other factors related to the formation of hydrocephalus include GCS score (*p* = 0.002) and Hunt Hess grade (*p* < 0.001), indicating that the severity of the condition at admission is also related to the formation of hydrocephalus. From Table 4, it can be seen that HH grading and postoperative cerebrospinal fluid drainage an independent influencing factors for the formation of hydrocephalus in patients with intraventricular hemorrhage. The higher the HH rating at admission, the poorer the patient’s state of consciousness. The reasons for poor consciousness may be related to many factors, but acute hydrocephalus is the one that cannot be ignored. In this study, postoperative cerebrospinal fluid drainage (OR = 0.110 [0.036–0.336], *p* < 0.001) was significantly helpful in reducing the formation of chronic hydrocephalus, effectively clearing blood mixed in cerebrospinal fluid and blood degradation products, thereby improving patient prognosis. Previous research results have also confirmed this, and the safety and effectiveness of CSF drainage from the lumbar cistern through lumbar drainage or continuous lumbar puncture (LP) have been confirmed for patients with mild HH levels at admission (28–30). For low-grade aSAH patients presenting with acute hydrocephalus, CSF drainage through ventricular drainage (EVD) has been proven to be necessary, effective, and safe (28). However, the actual advantages of early lumbar puncture cerebrospinal fluid drainage compared to EVD in aSAH patients still need to be determined, which hinders its widespread application. Based on the results of the second analysis in this study, we believe that for patients with intraventricular hemorrhage, regardless of the presence of acute hydrocephalus or increased intracranial pressure, cerebrospinal fluid drainage (at least lumbar puncture cerebrospinal fluid drainage) should be performed, which helps to reduce the risk of chronic hydrocephalus in patients.

**Table 4:** Multivariable model for factors influencing the formation of hydrocephalus in patients with intraventricular hemorrhage.

Variable	OR	95%CI	*P*-value
Female	0.415	0.153–1.124	0.084
GCS score	1.122	0.785–1.604	0.527
Hunt Hess grade	3.362	1.146–9.863	**0.027**
External cerebrospinal fluid	0.110	0.036–0.336	**<0.001**

The values in bold are statistically significant (*P* < 0.05). GCS, Glasgow Coma Scale; OR, Odds Ratio; CI, confidence interval.

The third analysis compared lumbar drainage with extraventricular drainage in patients with intraventricular hemorrhage. As shown in Table 5, the admission condition of patients receiving external ventricular drainage is more severe than that of patients receiving lumbar drainage (GCS score, Hunt Hess grade), because the choice of drainage method is determined by the attending neurosurgery physician, mainly depending on the patient’s intracranial pressure and whether there is acute hydrocephalus. For patients with acute hydrocephalus before surgery, external ventricular drainage is used, which can increase intracranial pressure, However, patients without acute hydrocephalus often use lumbar puncture for continuous drainage. We found that in univariate analysis, there was no statistically significant difference in the effectiveness of these two drainage methods in reducing the formation of chronic hydrocephalus in patients with intraventricular hemorrhage, and there was no significant difference in postoperative complications such as intracranial infection and DCI. However, when we included possible influencing factors and cerebrospinal fluid drainage methods in a multivariate logistic regression model, we found significant differences in GCS scores and the formation of chronic hydrocephalus between the two groups of patients at admission. The research results of Adams et al. (31) show that EVD, acute hydrocephalus upon admission, meningitis, and age are independent risk factors for the formation of hydrocephalus. In our study, the third analysis used a logistic regression model to adjust for the effects of smoking, admission GCS score and HH grade, and the formation of chronic hydrocephalus. The results showed that there was a significant difference between the two groups of patients in GCS score and the formation of chronic hydrocephalus. We expected the difference in GCS score, as patients who used EVD drainage developed acute hydrocephalus before surgery. Compared to patients with continuous lumbar drainage, their condition at admission is more severe and their condition is worse (Table 6). EVD and lumbar puncture drainage were not statistically significant in univariate analysis, but when we used logistic regression models to control for the effects of other factors, we found significant differences in the formation and drainage methods of chronic hydrocephalus. In our study, external ventricular drainage was superior to continuous lumbar drainage. Among the 15 patients who underwent external ventricular drainage, 4 patients developed chronic hydrocephalus, and among the 45 patients who underwent lumbar puncture drainage, 19 patients developed chronic hydrocephalus (26.7% vs. 42.2%). The reason for this situation may be that some patients undergoing lumbar puncture drainage already have acute hydrocephalus before the surgery, but the imaging manifestations are not obvious, which can affect the judgment of neurosurgeons. Alternatively, acute hydrocephalus may have occurred during the surgery. However, even so, combining our first, second, and third analyses, it is necessary to perform postoperative cerebrospinal fluid drainage in patients with aSAH who have combined IVH, which can significantly reduce the formation of chronic hydrocephalus. The method of cerebrospinal fluid drainage still deserves further research. There are still omissions in determining the drainage method based on imaging and clinical manifestations at admission. We hope to have a more effective and simple judgment method for neurosurgeons to quickly evaluate which cerebrospinal fluid drainage method patients should adopt.

**Table 5:** Comparison of baseline data on chronic hydrocephalus in patients with different postoperative drainage methods.

	Cerebrospinal fluid drainage		
Characteristic	LD (*n* = 45)	EVD (*n* = 15)	*P*-value
Age, years, mean (SD)	63.2 (10.0)	59.6 (9.2)	0.231
Female, *n* (%)	29 (64.4)	6 (40.0)	0.096
Smoking history, *n* (%)	1 (2.2)	4 (26.7)	**0.012**
History of drinking alcohol, *n* (%)	4 (8.9)	3 (20.0)	0.351
Hypertension, *n* (%)	33 (73.3)	10 (66.7)	0.620
Heart disease, *n* (%)	3 (6.7)	0 (0)	0.566
Diabetes, *n* (%)	6 (13.3)	1 (6.7)	0.668
History of aSAH, *n* (%)	2 (4.4)	0 (0)	0.559
GCS score, mean (SD)	12.3 (3.3)	7.3 (4.4)	**<0.001**
Hunt Hess grade, mean (SD)	2.2 (1.2)	3.8 (1.2)	**<0.001**
Aneurysm location			0.400
Anterior circulation, *n* (%)	40 (88.9)	12 (80.0)	
Posterior circulation, *n* (%)	5 (11.1)	3 (20.0)	
Aneurysm size (mm), mean (SD)	5.5 (1.8)	5.3 (1.5)	0.625
Aneurysm treatment time			0.496
24 (h), *n* (%)	37 (82.2)	12 (80.0)	
24–72 (h), *n* (%)	5 (11.1)	3 (20.0)	
>72 (h), *n* (%)	3 (6.7)	0 (0)	
Pulmonary infection, *n* (%)	22 (48.9)	11 (73.3)	0.137
Intracranial infection, *n* (%)	10 (22.2)	4 (26.7)	0.724
DCI, *n* (%)	3 (6.7)	1 (6.7)	0.742
Hydrocephalus	19 (42.2)	4 (26.7)	0.283
Length of stay, mean (SD)	17.4 (13.3)	20.7 (16.4)	0.444

The value in bold indicates *p* < 0.05. aSAH, aneurysmal subarachnoid hemorrhage; IVH, intraventricular hemorrhage; SD, standard deviation; EVD, external ventricular drainage; LD, lumbar drainage; DCI, delayed cerebral ischemia; GCS, Glasgow Coma Scale. Divide patients into two groups based on the presence or absence of the cerebrospinal fluid drainage method. Groups were compared using the χ^2^ test, Fisher exact test, or Student *t*-test.

**Table 6:** Multivariate logistic regression model adjustment for inter-group differences in patients with different methods of cerebrospinal fluid drainage.

Variable	OR	95%CI	*P*-value
Smoking history	0.608	0.053–7.020	0.690
GCS score	0.484	0.259–0.901	**0.022**
Hunt Hess grade	0.603	0.133–2.724	0.510
Hydrocephalus	0.010	0.001–0.207	**0.003**

The values in bold are statistically significant (*P* < 0.05). GCS, Glasgow Coma Scale; OR, Odds Ratio; CI, confidence interval.

Based on the results of these three items, we believe that IVH patients should actively undergo cerebrospinal fluid drainage after surgery. The selection of both drainage methods should be based on the severity of the patient’s condition at admission, combined with the symptoms of intracranial hypertension, and whether there is acute hydrocephalus formation. For mild patients (Hunt Hess grade I–III) without acute hydrocephalus, we recommend lumbar drainage. Although our third analysis found no significant difference in the formation of hydrocephalus between the two drainage methods in univariate analysis, significant differences were still observed in the multivariate logistic regression model after adjusting for the effects of other factors. However, external ventricular drainage surgery itself causes significant damage to brain tissue, carries the risk of puncture tract bleeding, and there is a risk of displacement of the drainage tube that requires re-catheterization, which undoubtedly increases the risk of intracranial infection (32, 33). For critically ill patients (Hunt Hess grade IV–V), we recommend performing extracerebral drainage, which can significantly reduce the generation of chronic hydrocephalus and quickly drain bloody cerebrospinal fluid to relieve intracranial pressure, reduce the occurrence of acute hydrocephalus, discharge bloody cerebrospinal fluid, reduce the stimulation of blood red protein breakdown products on blood vessels, and reduce the occurrence of vasospasm (34). For patients between these two situations, we also recommend EVD drainage, which may benefit some potential patients.

### Limitations

Potential limitations to our retrospective review include those that are inherent to all retrospective analyses. Additionally, whether the cerebrospinal fluid drainage and which drainage method is adopted is determined by the attending neurosurgeon, with no standard regimen or dosage. Finally, only 15 patients were administered EVD. This limited the statistical power of our study and our ability to study associations in additional outcome subanalyses (e.g., infections, hemorrhagic complications, and in-hospital mortality).

## Conclusion

IVH is an independent risk factor for patients developing chronic hydrocephalus. All aSAH patients with IVH should undergo cerebrospinal fluid drainage, which can significantly reduce the production of postoperative chronic hydrocephalus. For mild patients without acute hydrocephalus, continuous lumbar drainage should be actively performed, while for severe patients or patients who have already developed acute hydrocephalus, external ventricular drainage should be actively performed. For patients with conditions ranging from mild to severe, we recommend performing ventricular drainage, which may benefit some potential patients.

## Data availability statement

The raw data supporting the conclusions of this article will be made available by the authors, without undue reservation.

## Ethics statement

The studies involving humans were approved by Ethics Committee of the Affiliated Zhuzhou Hospital of Xiangya Medical College, Central South University. The studies were conducted in accordance with the local legislation and institutional requirements. Written informed consent for participation was not required from the participants or the participants’ legal guardians/next of kin in accordance with the national legislation and institutional requirements.

## Author contributions

BW: Data curation, Formal analysis, Methodology, Supervision, Writing-manuscript & editing. YZ: Data revalidation, Formal re-analysis, Methodology, Manuscript revision, Respond to reviewers’comments. HF: Data curation, Methodology, Formal analysis. ZL: Methodology, Formal analysis. WW: Methodology. ZC: Validation. YY: Validation. WY: Validation. WL: Conceptualization, Funding acquisition.

## References

[1] Catapano JS Zabramski JM Baranoski JF Brigeman S Morgan CD Hendricks BK. The prognostic significance of a cast fourth ventricle in ruptured aneurysm patients with intraventricular hemorrhage in the Barrow ruptured aneurysm trial (BRAT). Neurosurgery. (2019) 85:E275–83. doi: 10.1093/neuros/nyy493, PMID: 30476225

[2] Connolly ES Rabinstein AA Carhuapoma JR Derdeyn CP Dion J Higashida RT. Guidelines for the management of aneurysmal subarachnoid hemorrhage: a guideline for healthcare professionals from the American Heart Association/american Stroke Association. Stroke. (2012) 43:1711–37. doi: 10.1161/STR.0b013e3182587839 22556195

[3] Dorai Z Hynan LS Kopitnik TA Samson D. Factors related to hydrocephalus after aneurysmal subarachnoid hemorrhage. Neurosurgery. (2003) 52:763–71. doi: 10.1227/01.NEU.0000053222.74852.2D 12657171

[4] Dehdashti AR Rilliet B Rufenacht DA de Tribolet N. Shunt-dependent hydrocephalus after rupture of intracranial aneurysms: a prospective study of the influence of treatment modality. J Neurosurg. (2004) 101:402–7. doi: 10.3171/jns.2004.101.3.0402, PMID: 15352596

[5] Sheehan JP Polin RS Sheehan JM Baskaya MK Kassell NF. Factors associated with hydrocephalus after aneurysmal subarachnoid hemorrhage. Neurosurgery. (1999) 45:1120–8. doi: 10.1097/00006123-199911000-00021 10549928

[6] Vale FL Bradley EL Fisher WS 3rd. The relationship of subarachnoid hemorrhage and the need for postoperative shunting. J Neurosurg. (1997) 86:462–6. doi: 10.3171/jns.1997.86.3.0462, PMID: 9046303

[7] Varelas P Helms A Sinson G Spanaki M Hacein-Bey L. Clipping or coiling of ruptured cerebral aneurysms and shunt-dependent hydrocephalus. Neurocrit Care. (2006) 4:223–8. doi: 10.1385/NCC:4:3:223 16757827

[8] Kwon JH Sung SK Song YJ Choi HJ Huh JT Kim HD. Predisposing factors related to shunt-dependent chronic hydrocephalus after aneurysmal subarachnoid hemorrhage. J Korean Neurosurg Soc. (2008) 43:177–81. doi: 10.3340/jkns.2008.43.4.177, PMID: 19096639 PMC2588257

[9] Tapaninaho A Hernesniemi J Vapalahti M Niskanen M Kari A Luukkonen M. Shunt-dependent hydrocephalus after subarachnoid haemorrhage and aneurysm surgery: timing of surgery is not a risk factor. Acta Neurochir. (1993) 123:118–24. doi: 10.1007/BF01401866 8237488

[10] Kuo LT Huang AP. The pathogenesis of hydrocephalus following aneurysmal subarachnoid hemorrhage. Int J Mol Sci. (2021) 22:5050. doi: 10.3390/ijms22095050, PMID: 34068783 PMC8126203

[11] O’Kelly CJ Kulkarni AV Austin PC Urbach D Wallace MC. Shunt-dependent hydrocephalus after aneurysmal subarachnoid hemorrhage: incidence, predictors, and revision rates. J Neurosurg. (2009) 111:1029–35. doi: 10.3171/2008.9.JNS08881, PMID: 19361256

[12] Van Gijn J Kerr RS Rinkel GJ. Subarachnoid haemorrhage. Lancet. (2007) 369:306–18. doi: 10.1016/S0140-6736(07)60153-6 17258671

[13] Duan F Wang G Ma X Zhao Y Xu X Dong F. A controlled study of continuous lumbar drainage of fluid and lumbar puncture drainage for aneurysmal SAH after intracranial aneurysm clipping. J Healthc Eng. (2021) 2021:1–8. doi: 10.1155/2021/2827493 PMC8397562 34457216

[14] Dey M Jaffe J Stadnik A Awad IA. External ventricular drainage for intraventricular hemorrhage. Curr Neurol Neurosci Rep. (2012) 12:24–33. doi: 10.1007/s11910-011-0231-x, PMID: 22002766 PMC6777952

[15] Chung DY Mayer SA Rordorf GA. External ventricular drains after subarachnoid hemorrhage: is less more? Neurocrit Care. (2018) 28:157–61. doi: 10.1007/s12028-017-0443-2, PMID: 28929378 PMC5858985

[16] Bhattacharjee S Rakesh D Ramnadha R Manas P. Subarachnoid hemorrhage and hydrocephalus. Neurol India. (2021) 69:S429–33. doi: 10.4103/0028-3886.332266, PMID: 35102999

[17] Hochstetler A Raskin J Blazer-Yost BL. Hydrocephalus: historical analysis and considerations for treatment. Eur J Med Res. (2022) 27:168. doi: 10.1186/s40001-022-00798-6 36050779 PMC9434947

[18] Jartti P Karttunen A Isokangas JM Jartti A Koskelainen T Tervonen O. Chronic hydrocephalus after neurosurgical and endovascular treatment of ruptured intracranial aneurysms. Acta Radiol. (2008) 49:680–6. doi: 10.1080/02841850802050754, PMID: 18568561

[19] de Oliveira JG Beck J Setzer M Gerlach R Vatter H Seifert V. Risk of shunt-dependent hydrocephalus after occlusion of ruptured intracranial aneurysms by surgical clipping or endovascular coiling: a single-institution series and meta-analysis. Neurosurgery. (2007) 61:924–34. doi: 10.1227/01.neu.0000303188.72425.24 18091269

[20] Quigley M. Risk of shunt-dependent hydrocephalus after occlusion of ruptured intracranial aneurysms by surgical clipping or endovascular coiling: a single-institution series and meta-analysis. Neurosurgery. (2008) 63:E1209. doi: 10.1227/01.NEU.0000315869.57200.64 19057295

[21] Gruber A Reinprecht A Bavinzski G Czech T Richling B. Chronic shunt-dependent hydrocephalus after early surgical and early endovascular treatment of ruptured intracranial aneurysms. Neurosurgery. (1999) 44:503–9. doi: 10.1097/00006123-199903000-00039, PMID: 10069587

[22] Nguyen HS Li L Patel M Kurpad S Mueller W. Radiodensity of intraventricular hemorrhage associated with aneurysmal subarachnoid hemorrhage may be a negative predictor of outcome. J Neurosurg. (2018) 128:1032–6. doi: 10.3171/2016.11.JNS152839, PMID: 28474990

[23] Czorlich P Schweingruber N Göttsche J Mader MM Westphal M. Acute low-pressure hydrocephalus in aneurysmal subarachnoid hemorrhage. Neurosurg Focus. (2023) 54:E5. doi: 10.3171/2023.1.FOCUS22639, PMID: 37004138

[24] Asada R Nakatsuka Y Kanamaru H Kawakita F Fujimoto M Miura Y. Higher plasma Osteopontin concentrations associated with subsequent development of chronic shunt-dependent hydrocephalus after aneurysmal subarachnoid hemorrhage. Transl Stroke Res. (2021) 12:808–16. doi: 10.1007/s12975-020-00886-x 33423213

[25] Brisman JL Berenstein A. Factors related to hydrocephalus after aneurysmal subarachnoid hemorrhage. Neurosurgery. (2004) 54:1031. doi: 10.1227/01.NEU.0000117123.32806.F9 15088618

[26] Graff-Radford NR Torner J Adams HP Kassell NF. Factors associated with hydrocephalus after subarachnoid hemorrhage. A report of the cooperative aneurysm study. Arch Neurol. (1989) 46:744–52. doi: 10.1001/archneur.1989.00520430038014 2742543

[27] Hao X Wei D. The risk factors of shunt-dependent hydrocephalus after subarachnoid space hemorrhage of intracranial aneurysms. Medicine. (2019) 98:e15970. doi: 10.1097/MD.0000000000015970, PMID: 31277089 PMC6635240

[28] Hasan D Vermeulen M Wijdicks EF Hijdra A van Gijn J. Management problems in acute hydrocephalus after subarachnoid hemorrhage. Stroke. (1989) 20:747–53. doi: 10.1161/01.STR.20.6.747, PMID: 2728040

[29] al-Tamimi YZ Bhargava D Feltbower RG Hall G Goddard AJP Quinn AC. Lumbar drainage of cerebrospinal fluid after aneurysmal subarachnoid hemorrhage: a prospective, randomized, controlled trial (LUMAS). Stroke. (2012) 43:677–82. doi: 10.1161/STROKEAHA.111.625731 22282887

[30] Liang C Yang L Guo S. Serial lumbar puncture reduces cerebrospinal fluid (CSF) infection during removal of hemorrhagic CSF in aneurysmal subarachnoid hemorrhage after endovascular coiling. J Biomed Res. (2018) 32:305–10. doi: 10.7555/JBR.32.20170028, PMID: 30047495 PMC6117608

[31] Adams H Ban VS Leinonen V Aoun SG Huttunen J Saavalainen T. Risk of shunting after aneurysmal subarachnoid hemorrhage: a collaborative study and initiation of a consortium. Stroke. (2016) 47:2488–96. doi: 10.1161/STROKEAHA.116.013739 27633019

[32] Pandit AS Palasz J Nachev P Toma AK. Mechanical complications of external ventricular and lumbar drains. World Neurosurg. (2022) 166:e140–54. doi: 10.1016/j.wneu.2022.06.127 35787961

[33] Palasz J D’Antona L Farrell S Elborady MA Watkins LD Toma AK. External ventricular drain management in subarachnoid haemorrhage: a systematic review and meta-analysis. Neurosurg Rev. (2022) 45:365–73. doi: 10.1007/s10143-021-01627-w, PMID: 34448080

[34] Haldrup M Miscov R Mohamad N Rasmussen M Dyrskog S Simonsen CZ. Treatment of intraventricular hemorrhage with external ventricular drainage and fibrinolysis: a comprehensive systematic review and Meta-analysis of complications and outcome. World Neurosurg. (2023) 174:183–196.e6. doi: 10.1016/j.wneu.2023.01.021, PMID: 36642373

